# Infectivity and genes differentially expressed between young and aging theront cells of the marine fish parasite *Cryptocaryon irritans*

**DOI:** 10.1371/journal.pone.0238167

**Published:** 2020-08-28

**Authors:** Hongshu Chi, Michael Goldstein, Angel Pichardo, Zung-Hang Wei, Wei-Jen Chang, Hui Gong

**Affiliations:** 1 Biotechnology Institute, Fujian Academy of Agricultural Sciences, Fuzhou, Fujian, China; 2 Department of Biology, Hamilton College, Clinton, New York, United States of America; 3 School of Mechanics and Engineering Science, Zhengzhou University, Zhengzhou, Henan, China; 4 State Key Laboratory of Large Yellow Croaker Breeding, Ningde, Fujian, China; 5 School of Marine Sciences, Ningbo University, Ningbo, Zhejiang, China; Texas A&M Health Science Center, UNITED STATES

## Abstract

The ciliated protozoan *Cryptocaryon irritans* infects a wide range of marine fish and causes the highly lethal white spot disease. This parasite possesses three morphologically and physiologically distinct life stages: an infectious theront, a parasitic trophont, and an asexually reproductive tomont. In the past few years, several attempts have been made to help elucidate how C. irritans transforms from one stage to another using transcriptomic or proteomic approaches. However, there has been no research studying changes in transcription profiles between different time points of a single *C*. *irritans* life stage—the development of this parasite. Here we use RNA-seq and compare gene expression profiles of theront cells collected by 1 and 10 hrs after they emerged from tomonts. It has been shown that infectivity of theront cells declines 6–8 hours post-emergence, and we used this characteristic as a physiological marker to confirm the aging of theront cells. We identified a total of 41 upregulated and 90 downregulated genes that were differentially expressed between young and aging theront cells. Using Blast2Go to further analyze functions of these genes, we show that genes related to energy production are downregulated, but quite surprisingly many genes involved in transcription/translation processes are upregulated. We also show that expression of all nine detectable agglutination/immobilization antigen genes, with great sequence divergence, is invariably downregulated. Functions of other differentially expressed genes and indications are also discussed in our study.

## Introduction

Unicellular eukaryotes often need to respond dynamically to changes in their living environments. The ability to meet conditional demands promptly is particularly important for parasitic protozoans because their physiological priorities might be different before, during, and after establishing successful infections on hosts. Regulation of genes whose products are involved in metabolic pathways could be even more dynamic and complicated in parasites where dramatic morphological changes occur during their life cycles. One such example is the prostome ciliate *Cryptocaryon irritans*, an obligate parasite that causes the highly lethal white spot disease in marine fish.

**Fig 1:**
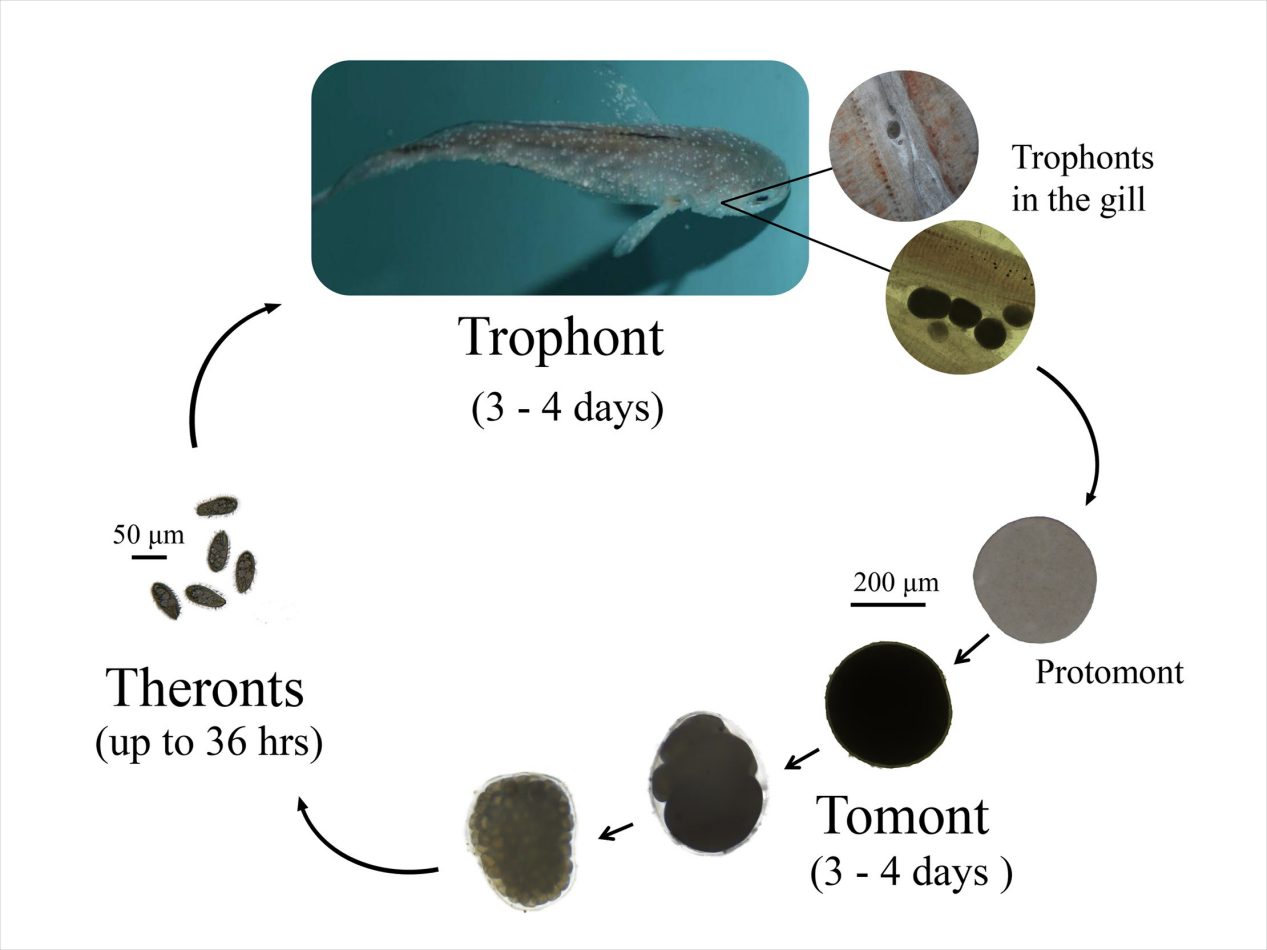
The life cycle of *C*. *irritans*.

The life cycle of *C*. *irritans* consists of three morphologically and functionally distinct stages: a parasitic trophont stage, an encysting and asexually reproducing tomont stage, and an infectious theront stage (Fig 1). The trophont cells develop from theront cells that successfully infect host fish, feed in the epithelial layer of the hosts and progressively grow into the large, characteristic white spots. After 3–4 days, trophont cells mature and leave the host fish, become encysted on a flat surface as tomont cells, and undergo rounds of asexual divisions to give rise to the free-swimming, infectious theront cells [1].

*C*. *irritans* is capable of infecting a wide range of marine fish, and has been found in nearly all major seas [1] with high genetic diversity [2]. Moreover, *C*. *irritans* infections are highly lethal and often cause huge economic losses when outbreaks occur in mariculture. Scientists in China, Japan, and Malaysia have made numerous efforts to find ways to help curb *C*. *irritans* outbreaks. Most research projects focus on understanding host fish’s immune responses during *C*. *irritans* infections [3–19], and on testing antigens for effective immunizations in fish [20–23]. However, little research has been dedicated to study gene expression in *C*. *irritans*: Lokanathan and colleagues sequenced more than 5,000 cDNA clones from *C*. *irritans* tomont cells in a pioneering study [24]; Mai *et al*. compared proteins differentially expressed in the three key life stages of *C*. *irritans* [25], and Mo *et al*. followed up with transcriptomic profiling using next generation sequencing [26]. Results derived from these studies not only allow for further understanding of the biology of this parasite at the molecular level, but also provide valuable information for comparative work. None of these studies, however, compared genes differentially expressed at different time points in one life stage of *C*. *irritans*.

Theronts, the infectious unit, can survive in saltwater for one to two days [27, 28]. However, it has been noted that theront infectivity declined significantly 6–8 hours post-excystment [29, 30]. Both Yoshinaga and Dickerson [29] and Burgess and Matthers [30] showed that 10 hours post-excystment theronts were not able to complete successful infection and mature into trophont exit. We suspect that transcription profiles of young and aging may have changed and perform both infectivity assays and RNA-seq to help identify genes that significantly change their expression levels between 1 and 10 hours after theront formation. The information gathered from this study provides insight into the molecular changes *C*. *irritans* goes through during its theront state.

## Materials and methods

### *C*. *irritans* and the infectivity assay

*C*. *irritans* were obtained from infected cage culture *Lates calcarifer* from Ningde-Sandu’ao, Fujian, China. The parasite was maintained and propagated in the lab using the same host fish species in seawater (27‰) at room temperature with constant aeration (1–2 L/min). In the infectivity assay, theronts were collected within one hour window after they started massively emerging from tomont cells. After sitting for 1 hr and 10 hrs theronts from the top layer were counted and were used to infect *L*. *calcarifer* at a nonlethal dosage of 2,000 theronts per fish. Fifteen *L*. *calcarifer* (20 +/- 1 g), divided into three groups, were used in each condition, and they were transferred to new aquaria 48 hrs post infection. Trophonts released from the infected fish, primarily 72–96 hrs post infection, were collected and the numbers of trophonts were counted. The protocol of using fish was approved by the Institutional Animal Care and Use Ethics Committee of Fujian Academy of Agricultural Science (protocol number 0007055002).

### RNA isolation and RNA-sequencing

Tomonts that gave rise to the theront cells used for RNA-seq were collected from three replicates of five infected *L*. *calcarifer*. Tomonts from each replicate were allowed to independently develop into theronts, which were then harvested 1 hr and 10 hrs post-excystment. Total RNA was extracted using the RNeasy mini kit (Qiagen, Germantown, MD) following manufacturer’s protocols with DNase treatments. For RNA-seq, RNA was poly-A selected and six cDNA libraries (three each at 1 hr and 10 hrs) were constructed and sequenced at Genewiz (South Plainfield, NJ) using Illumina HiSeq 2500. RNA-seq data is deposited in GenBank’s Sequence Read Archive (SRA) under the accession number PRJNA600221.

### Differential expression gene detection

The Illumina 100-bp paired end raw reads were first aligned to a *C*. *irritans* transcriptome published by Lokanathan et al. (GenBank accession no.: GEEV00000000.1). This transcriptome was used because it was the only publicly available one for *C*. *irritans*, and showed a low level of contamination from fish (~ 15%) [24]. Gene expression values were determined by using the RSEM package [31], and differentially expressed genes (DEGs) were identified using the EBseq package [32]. The RSEM package uses bowtie2 [33] to help map short Illumina reads to a genome/transcriptome, and calculates gene expression values using a generative statistical model and associated inference methods when handling non-uniquely mapped reads [34]. EBseq uses an empirical Bayes hierarchical method to help detect differential expressed genes based on their expression values and we set a hard false discovery rate threshold at 0.05 [32]. When a change of expression of a gene carries a posterior probability equal or greater than 1—FDR (0.05 in this case) the change is deemed significant. From the list of genes that satisfy this EBseq statistical cutoff, we further set a 4.95 real fold-change threshold (see maintext). Both genes that were significantly and differentially regulated were subjected to Blast2Go analyses [35, 36]

### Realtime quantitative PCR verification

Expressions of fourteen randomly selected genes were subjected to realtime quantitative RT-qPCR verification. Total RNA was reverse-transcribed into cDNA using the TaKaRa PrimeScript RT Reagent with gDNA Eraser (TaKaRa Biomedical Technology, Beijing, China), and real-time quantitative PCR was performed on an Applied Biosystems ViiA 7 Dx Real-Time PCR Instrument using TaKaRa SYBR Fast qPCR Mix. The expression of each gene at each of the two time points was assessed in triplicates. Quantification of gene expression was carried out by using the 2^-ΔΔCT^ method and the expression of 18S rRNA was used as the reference. Primer information can be found in Supporting Materials (S1 Table).

### Phylogenetic analyses

Nine different agglutination/immobilization antigen (I-antigen) genes were identified in the transcriptome published by Lokanathan et al. [24] using BLAST. These nucleotide sequences were conceptually translated into protein sequences using ciliate genetic code. Together with 18 other *C*. *irritans* I-antigen protein sequences downloaded from the NCBI protein database, these 27 sequences were aligned using T-Coffee [37], and their phylogenetic relationships were resolved using a maximum-likelihood method (PhyML) [38] with Dayhoff+G+F model determined by using Prottest [39] and 100 bootstrapping replicates to assess branch supports. Accession numbers of all 27 *C*. *irritans* I-antigen gene sequences are provided in Supporting Materials (S2 Table).

## Results

### *C*. *irritans* theront infectivity

**Fig 2:**
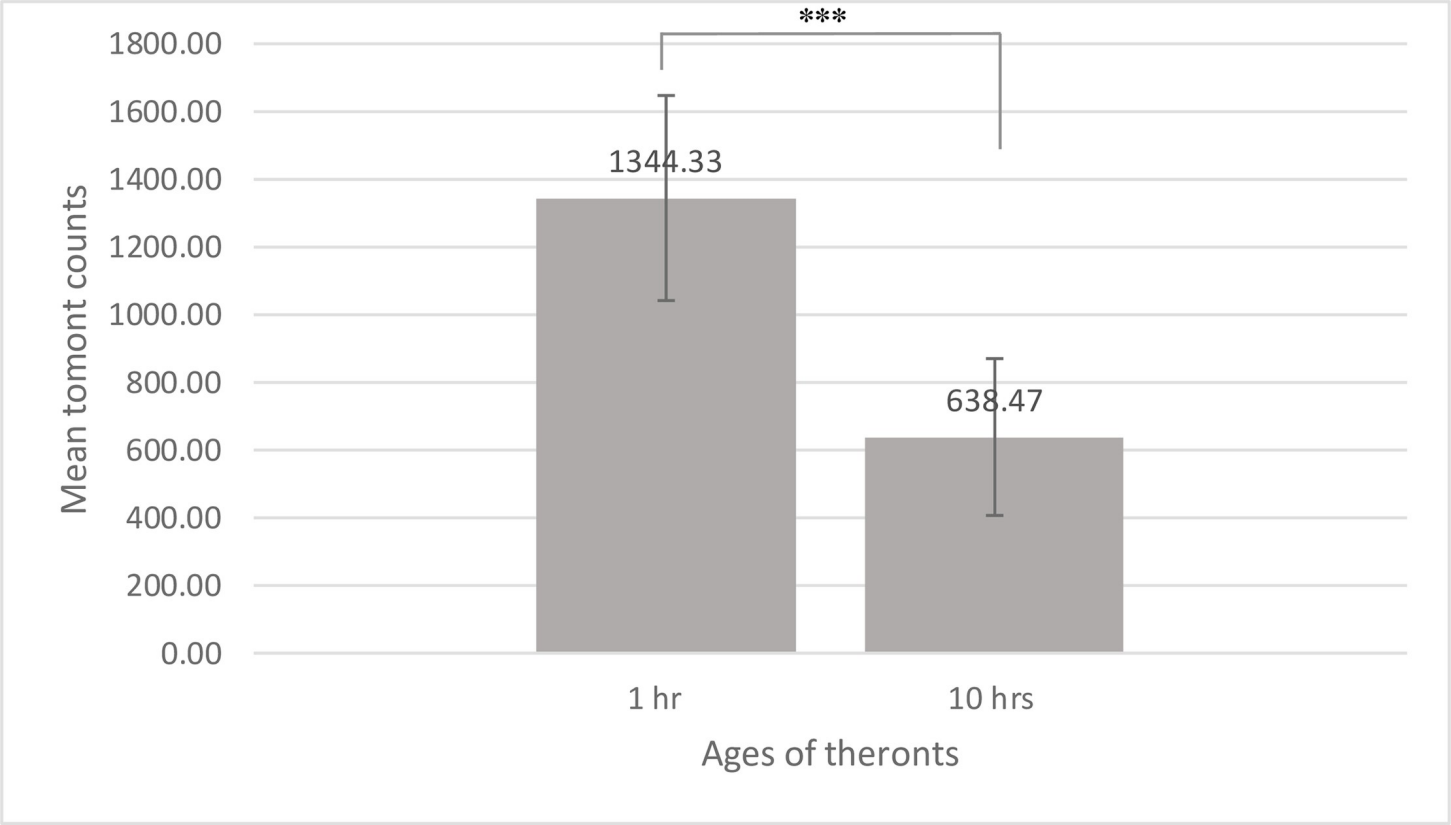
Infectivity of theronts collected from two different time points. Theronts collected from 1 hr and 10 hrs after they emerged from tomonts were used to infect 15 *L*. *calcarifer* for each time point. Successful infections that resulted in mature trophonts leaving host fish were measured by the numbers of tomonts collected from each infected fish. Mean tomont counts and standard deviations are provided for both time points (*t*-test, ***p < 0.001).

Infectivity is defined as the number of theronts establishing successful infections, developing fully into trophonts, and detaching from the host fish after trophonts mature. It is measured by the numbers of tomonts collected from infected fish. Two different ages of *C*. *irritans* theronts (1 hr and 10 hrs post-excystment) were used to infect *L*. *calcarifer* at a nonlethal dosage, and numbers of tomonts released from fish were counted. Our results show that theront infectivity declined significantly by more than 50% from 1 hr to 10 hrs after theronts excysted (***P < 0.001, Fig 2), and confirmed the aging of theronts. The trend is also consistent with observations made by Burgess and Matthews [30], and Yoshinaga and Dickerson [29]: both groups reported significant decline of infectivity from theronts collected 6 hours and on after they emerged from cysts.

### RNA-seq analyses

**Fig 3:**
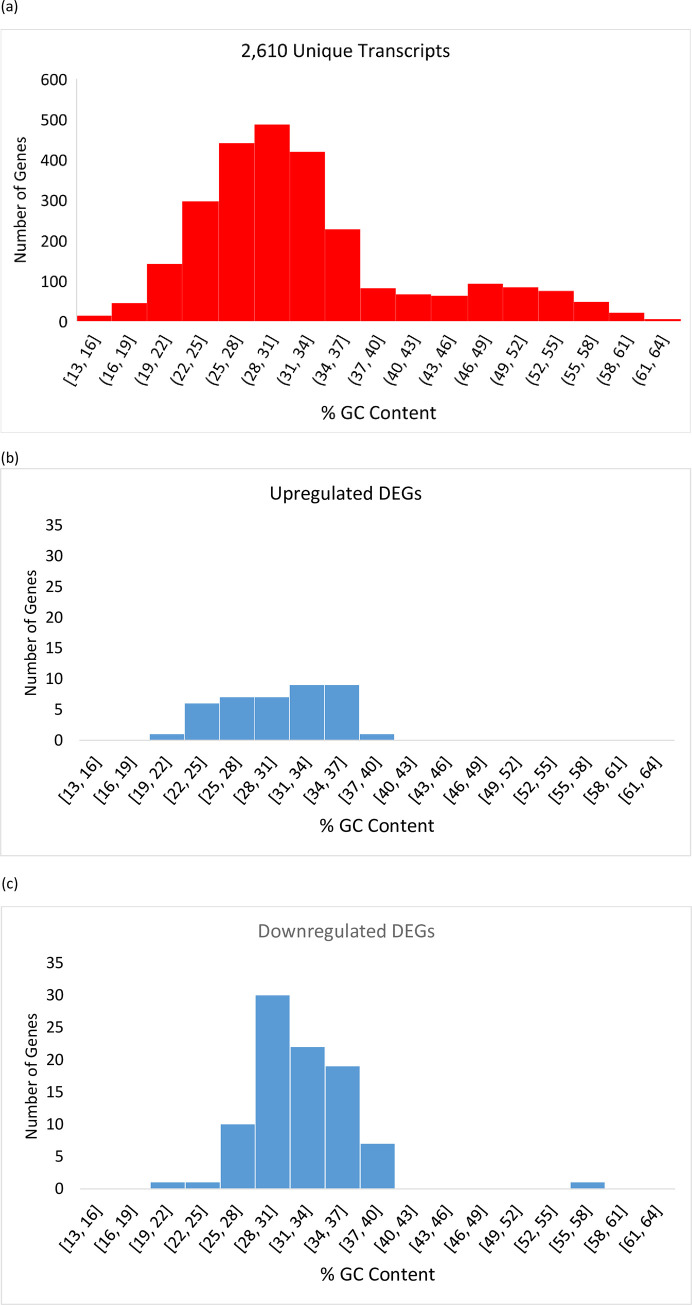
Histograms of GC percentages of (a) the 2,610 unique transcripts from *C*. *irritans* published by Lokanathan and colleagues [24], and (b) upregulated and (c) downregulated differentially expressed genes.

To explore changes in gene expression from 1 hr to 10 hrs post theront formation, we performed RNA-seq on *C*. *irritans* theront cells at both time points, with three replicates each. Six cDNA libraries (three from 1 hr and three from 10 hrs theront cells) were subjected to Illumina 100-bp paired-end sequencing which yielded a total of 412,760,652 reads (Table 1). Raw reads were aligned to a *C*. *irritans* transcriptome published by Lokanathan and colleagues which contains 1,806,497 base pairs in 2,610 unique transcripts [24]. Because a parasite’s transcriptome often contains contaminants from its host, we assessed the quality of the *C*. *irritans* transcriptome by using a GC plot: we created a GC content histogram of those 2,610 unique transcripts and the resulting plot shows a major peak between 28–31 percent and some minor contamination from species whose transcripts have a much higher GC peaking at around 49 percent (Fig 3A). Comparing high GC sequences (>42%) against protein sequences in the GenBank nr database using blastx showed that most of these contaminants derived from fish (S3 Table). There are 417 unique transcripts, or 15.98% of the transcriptome, whose GC percentages are higher than 42%. This number is very close to the percentage of fish contaminants (15%) that Lokanathan and colleagues estimated based on their BLAST results [24], suggesting that GC plots are effective here in separating contaminating sequences and that those unique transcripts with lower GC likely belong to *C*. *irritans*.

**Table 1:** Statistic of RNA-seq and alignments to a *C*. *irritans* transcriptome.

Libraries	No. of Raw Reads	No. of Uniquely Aligned Raw Reads	Percent Unique Transcripts with Aligned Reads	Average Coverage of the Transcriptome
1h_1	61,287,490	28,864,716 (47%)	55.82	1613.81
1h_2	54,030,814	15,920,478 (29%)	38.54	890.10
1h_3	65,867,604	19,814,560 (30%)	59.16	1107.82
10h_1	106,418,160	51,862,406 (49%)	64.71	2891.43
10h_2	69,725,670	12,015,048 (17%)	45.79	671.75
10h_5	55,430,914	7,965,194 (14%)	51.22	445.33

The six cDNA libraries yielded different numbers of reads ranging from approximately 54 to 106 million. However, regardless of the yields, reads from each library aligned well to the transcriptome: 38% to 64% of the unique transcripts from the transcriptome were aligned to at least one pair of raw reads, and average coverage of the transcriptome ranged from 445X to 2,891X (Table 1).

Gene expression values (FPKM) were calculated by using the RSEM package [31]. Plotting mean FPKM values from 1,858 genes that were expressed in either or both 1 hr and 10 hrs theronts shows that most genes did not change their expression levels significantly (R^2^ = 0.844, S1 Fig). In fact, between the top 100 highly expressed genes in 1 hr and 10 hrs theront, 77 genes were shared (S4 Table). Among the top 20 highly expressed genes, thirteen overlap between the 1 hour and 10 hour lists (Table 2). Many of these highly expressed genes are involved in translation (ribosomal RNA and proteins, and elongation factor 2). In the 1 hr samples transcripts from several agglutination/immobilization (I-antigen) genes were also abundant. Finally, the names of a few of the top 20 highly expressed genes from our samples also matched to those that were found highly expressed in another transcriptomic profiling of *C*. *irritans* theront cells (Table 2) [26], highlighting the consistency of the expression pattern in the theront stage of this parasite across different samples.

**Table 2:** Top 20 highly expressed genes in the 1 hr theront samples.

Gene ID	Blast Annotations	FPKM	Notes
gb|GEEV01000049.1|	rRNA	5,349,284.00	#
gb|GEEV01000189.1|	rRNA	5,211,654.00	#
gb|GEEV01000137.1|	no hit	1,824,867.00	#
gb|GEEV01000838.1|	translationally controlled protein	193,136.00	#^
gb|GEEV01000435.1|	MORN repeat protein	144,628.90	#^
gb|GEEV01000095.1|	agglutination/immobilization antigen	35,681.03	
gb|GEEV01000542.1|	heat shock protein 90	34,113.38	#^
gb|GEEV01000094.1|	agglutination/immobilization antigen	33,331.87	
gb|GEEV01000100.1|	ubiquitin from rockcod	31,150.00	#
gb|GEEV01000231.1|	cathepsin (protease)	23,830.75	#^
gb|GEEV01000392.1|	hypothetical protein	21,640.70	#
gb|GEEV01000279.1|	elongation factor 2	21,335.01	#^
gb|GEEV01000502.1|	f-1,6-bp aldolase	19,577.26	
gb|GEEV01000148.1|	ornithine decarboxylase antizyme	18,594.61	#
gb|GEEV01000074.1|	alpha-tubulin	18,356.32	
gb|GEEV01001835.1|	RNA binding domain protein	16,828.28	#
gb|GEEV01001419.1|	ornithine decarboxylase antizyme	12,748.21	#
gb|GEEV01000096.1|	agglutination/immobilization antigen	12,571.54	
gb|GEEV01000327.1|	nuclear migration protein	11,766.70	
gb|GEEV01000039.1|	agglutination/immobilization antigen	10,503.41	

# gene is also one of the top 20 highly expressed genes in 10 hrs theront samples

^ also highly expressed in theronts in Mo et al.

### Realtime quantitative PCR verification

**Fig 4:**
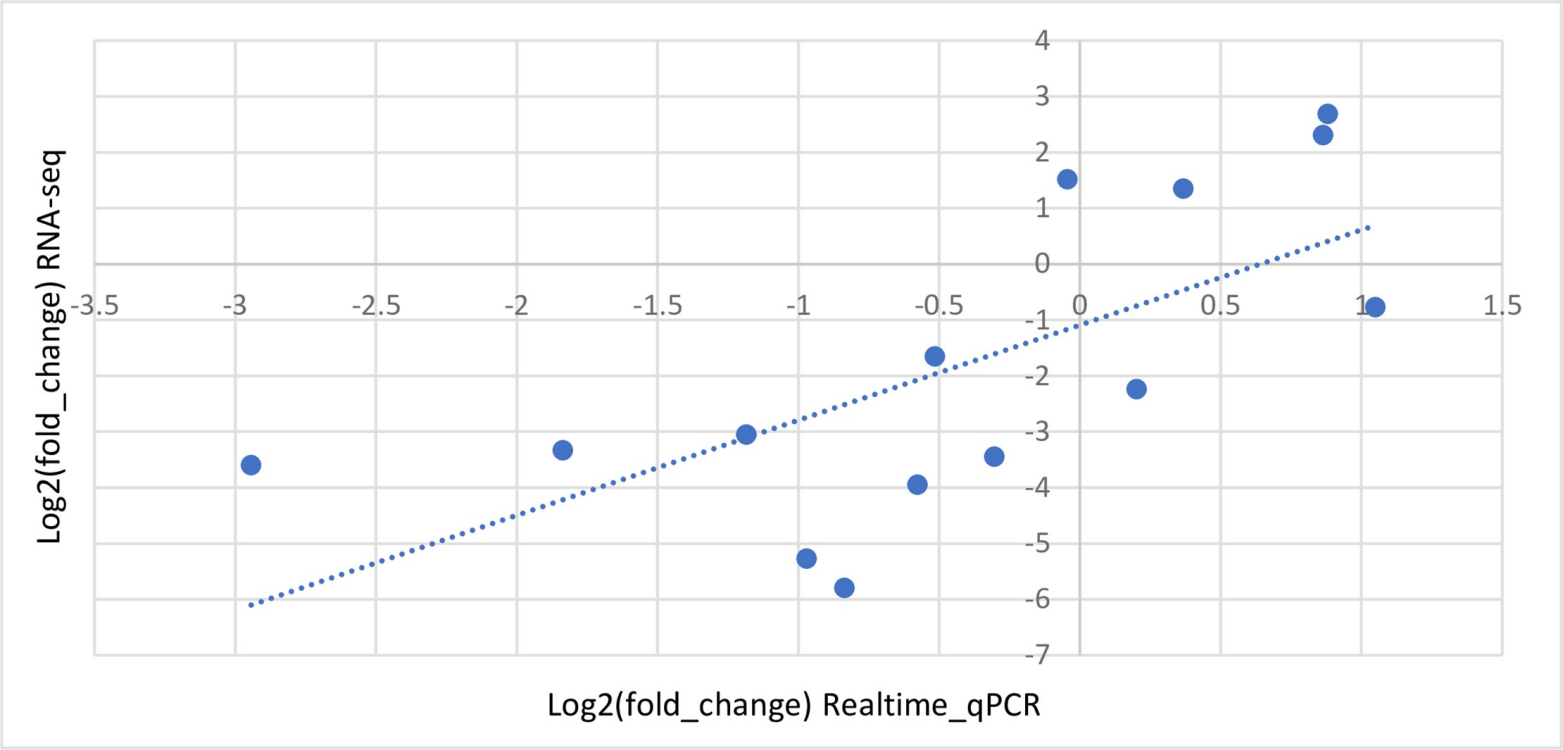
Correlation of changes in gene expression between RNA-seq data and realtime quantitative PCR results. Log base 2 values of fold changes in gene expression from 14 genes detected by using either of the methods were plotted (R^2^ = 0.68).

Expression of fourteen randomly selected genes were subjected to realtime quantitative PCR (RT-qPCR) verification. Among these fourteen genes, three out of five whose RNA-seq real fold changes were smaller than 3.0 (2^1.58^, upregulated) or greater than 0.20 (2^-2.32, downregulated) showed inconsistent results between RT-qPCR and RNA-seq (second and fourth quadrants, Fig 4). The other nine genes that showed greater fold changes in RNA-seq data, in contrast, showed consistent trends in RT-qPCR data (first and third quadrants, Fig 4). Using this information, we set a biological cutoff of 4.95 fold change when defining differentially expressed genes (see below).

### Differentially expressed genes

Differentially expressed genes (DEGs) were identified following two criteria: posterior probability (PPDE) greater than or equal to 0.95, and real fold changes in expression greater than 4.95 (upregulated genes) or smaller than 0.202 (downregulated genes). Using the statistical cutoff (PPDE > = 0.95) we were able to identify 98 upregulated and 131 downregulated genes that significantly changed their expression levels (S5 Table). By further applying the real fold change cutoff, we found 41 upregulated and 91 downregulated DEGs and histograms of GC percentages of these DEGs are shown in Fig 3B and 3C, respectively. Among these genes, only one (gb|GEEV01001602.1) had a high GC percentage at 57.8% (Fig 3C). BLAST search results, by virtue of a 100% identity and 96% query cover, showed that this DEG was indeed a contaminant from the host fish *L*. *calcarifer*, and was removed from further analyses. For the remaining 131 DEGs we ran a multitude of BLASTs on a random set of about 40 DEGs, and all of them either yielded no hits or corresponded with a ciliate origin (S6 Table).

**Fig 5:**
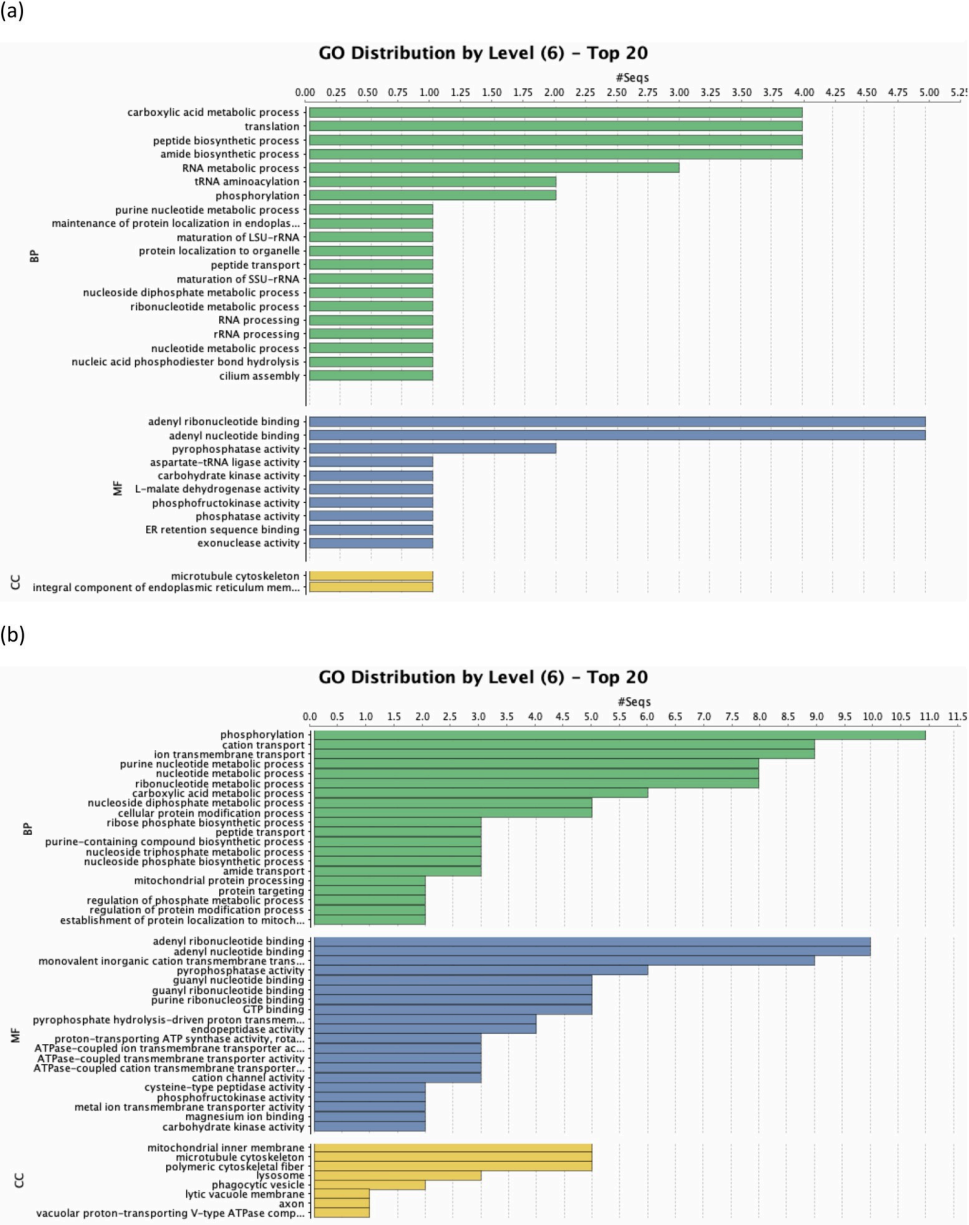
Level 6 GO term distributions among (a) upregulated and (b) downregulated differentially expressed genes.

We used Blast2GO [36] to help further parse out functions of genes whose expression levels changed significantly (PPDE > = 0.95, S7 Table) and of DEGs (PPDE > = 0.95 and real fold change > 4.95 or < 0.202, S8 Table). Genes that were upregulated in 10 hrs samples were mostly involved in transcription and translation processes, which included rRNA synthesis and translation (Fig 5A and S2 Fig). Many genes involved in cellular protein modification processes were also significantly upregulated in the 10 hrs samples (S2 Fig), however, only the expression level of a calmodulin kinase gene (GEEV01002117.1) passed the real fold change threshold (S7 Table). Noteworthy, the expression level of the Bax1 inhibitor gene, whose protein product has been shown to inhibit apoptosis/stress-signaling in multicellular eukaryotes and in yeasts (for review [40]), was also significantly elevated in 10 hrs samples (Table 3 and S8 Table).

**Table 3:** Expression levels of select sets of genes.

Gene ID	Description	Length	1hr_Mean	10hrs_Mean
gb|GEEV01000496.1|	inhibitor of apoptosis-promoting Bax1 protein	588	62.34	401.35
gb|GEEV01000109.1|	ATP synthase beta subunit precursor, putative	724	21.05	3.11
gb|GEEV01000613.1|	ATP synthase alpha subunit precursor, putative	837	59.40	2.54
gb|GEEV01002446.1|	ATP synthase beta subunit precursor, putative	662	73.25	4.74
gb|GEEV01000502.1|	fructose-bisphosphate aldolase	754	19577.26	1146.95
gb|GEEV01001025.1|	ATP-dependent 6-phosphofructokinase 3	696	508.47	41.95
gb|GEEV01001251.1|	pyruvate kinase	617	452.76	26.76
gb|GEEV01001825.1|	phosphofructokinase family protein, putative	679	150.62	22.96
gb|GEEV01000639.1|	Ubiquitin carboxyl-terminal hydrolase 14	742	8.73	1.02
gb|GEEV01001913.1|	Putative Ubiquitin carboxyl-terminal hydrolase	606	10.56	0.85
gb|GEEV01000697.1|	calpain-type cysteine protease DEK1	647	13.60	1.40
gb|GEEV01000803.1|	proteasome subunit beta type-4-like	687	41.58	6.11
gb|GEEV01001485.1|	cathepsin D-like	559	671.37	37.57
gb|GEEV01002259.1|	zinc carboxypeptidase superfamily protein	770	6.75	0.34
gb|GEEV01002287.1|	rhomboid protease	625	23.86	3.43
gb|GEEV01000732.1|	carboxylesterase	713	886.48	102.38
gb|GEEV01000740.1|	serine esterase, putative	750	16.93	3.23
gb|GEEV01000922.1|	putative acyl-CoA dehydrogenase	618	25.14	4.96
gb|GEEV01002074.1|	transmembrane protein, putative	586	0.78	18.02
gb|GEEV01001107.1|	phosphofructokinase family protein, putative	681	0.63	10.21
gb|GEEV01002192.1|	kinesin motor catalytic domain protein	656	80.24	15.86
gb|GEEV01000364.1|	tubulin beta, putative	690	2873.68	28.67
gb|GEEV01000229.1|	tubulin alpha chain	738	182.77	29.95
gb|GEEV01000263.1|	beta-tubulin, putative	1399	313.91	10.63
gb|GEEV01000291.1|	alpha-tubulin	659	412.88	64.08
gb|GEEV01000074.1|	tubulin alpha chain	1473	18356.32	1491.16
gb|GEEV01000007.1|	putative vacuolar ATP synthase subunit A	1223	233.55	21.39
gb|GEEV01000793.1|	putative vacuolar ATP synthase subunit d	685	212.43	8.88
gb|GEEV01001954.1|	vacuolar ATP synthase subunit e, putative	637	175.63	15.74
gb|GEEV01002596.1|	vacuolar ATP synthase subunit c, putative	732	466.95	31.14
gb|GEEV01000036.1|	agglutination/immobilization antigen	778	189.52	7.28
gb|GEEV01000037.1|	agglutination/immobilization antigen	1057	4690.16	491.87
gb|GEEV01000038.1|	agglutination/immobilization antigen	1108	5162.24	291.34
gb|GEEV01000039.1|	agglutination/immobilization antigen	1109	10503.41	682.22
gb|GEEV01000053.1|	agglutination/immobilization antigen	1115	1122.71	20.24
gb|GEEV01000054.1|	agglutination/immobilization antigen	1105	2054.51	132.70
gb|GEEV01000094.1|	agglutination/immobilization antigen	718	33331.87	4711.03
gb|GEEV01000096.1|	agglutination/immobilization antigen	1086	12571.54	1299.51
gb|GEEV01000095.1|	agglutination/immobilization antigen	1090	35681.03	4539.48

Genes with the GO term ATP-binding were among the most prominent in both significantly (sixteen) and differentially downregulated (ten) sets of genes (S2 Fig and Fig 5B). Further parsing these downregulated ATP-binding genes showed that some of them were involved in oxidative phosphorylation (Table 3 and S8 Table). In fact, many genes involved in other catabolic pathways that could lead to potential energy generation, i.e. glycolysis (four), proteolysis (seven) and lipid-breakdown (three), were also downregulated (Table 3 and S9 Table). These numbers would compare to one glycolytic pathway gene and one putative endopeptidase gene which were upregulated in 10 hrs theronts (Table 3). In addition, two sets of genes were downregulated in 10 hrs theront samples: six genes related to cytoskeleton (tubulin proteins and one kinesin motor domain protein) and four of the V-ATPase genes (Table 3).

Because genes related to transcription and translation were upregulated, we further investigated the expression of DNA-binding transcription factor genes (GO:0003700 and GO:0006355). Among 2,193 genes whose GC percentages were under 42% there were four annotated with the two GO terms (S10 Table). Two of the genes showed detectable levels of expression in both 1 hr and 10 hr samples, but not the other two. Neither of the two expressed genes changed their expression levels significantly (PPDE < 0.95, S10 Table). We also investigated genes annotated with GO:1903506 (regulation of nucleic-acid templated transcription) and/or GO:0010468 (regulation of gene expression). While all these genes showed detectable gene expression in both stages, their expression levels did not change significantly (S10 Table).

Finally, we observed that 8 different agglutination/immobilization antigen (I-antigen) genes, including three of the four from the top 20 highly expressed genes in 1 hr samples (Table 2), were significantly downregulated in 10 hrs theront cells. In fact, we identified a total of 9 I-antigen genes in the transcriptome published by Lokanathan and colleagues and expression of all nine went down in 10 hrs theront samples (Table 3). A table containing functional annotations and expression values of the 131 DEGs is provided in Supplementary Materials (S8 Table).

### Phylogenetic relationships among *C*. *irritans* I-antigen genes

**Fig 6:**
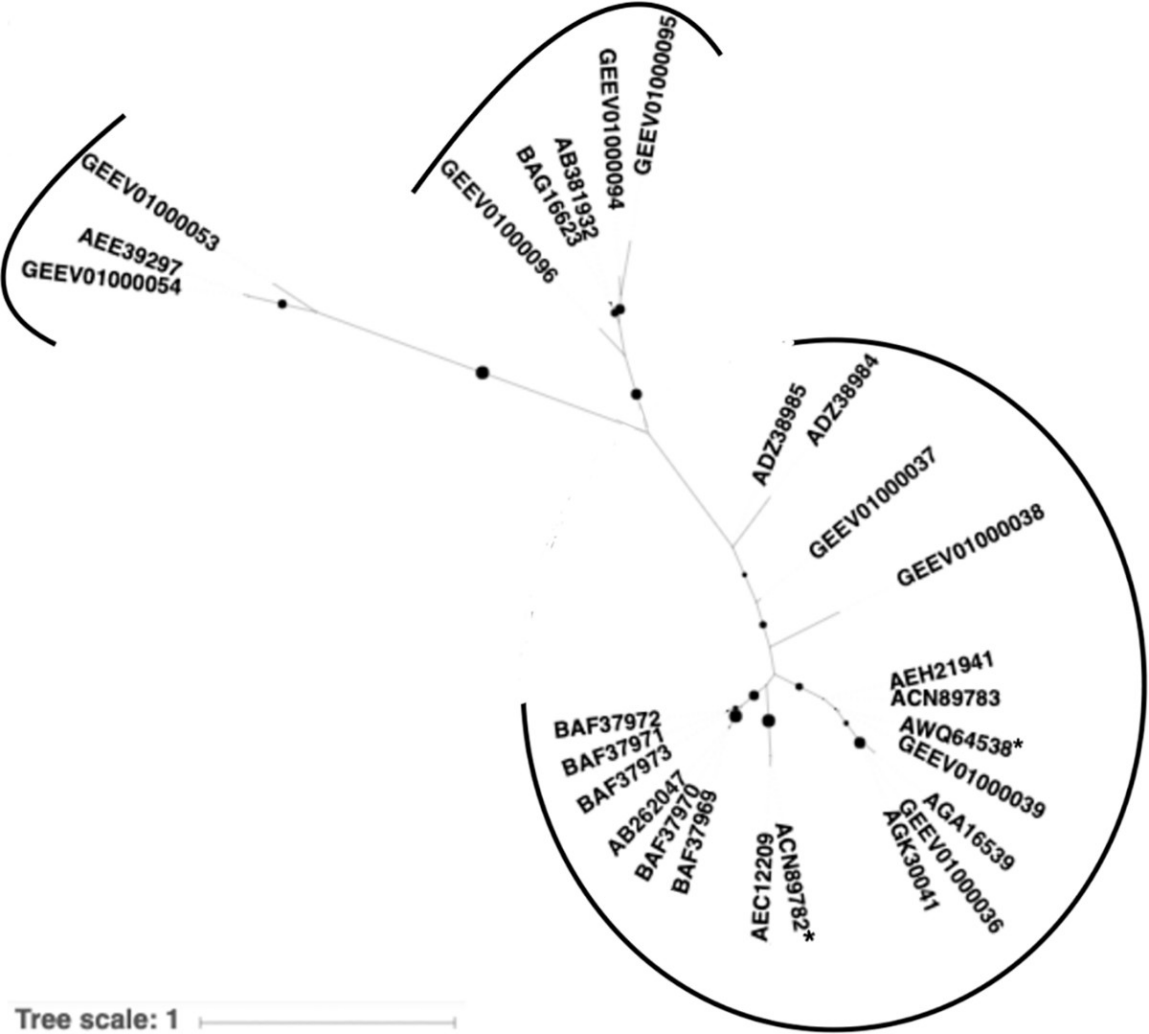
Maximum likelihood tree of 27 I-antigen sequences from *C*. *irritans*. Bootstrapping values are represented by size of black dots, with a cutoff of 70. Three distinct phylogenetic groups are indicated by black arcs, and sequences that have been used in vaccine developments are indicated with asterisks.

While compiling I-antigen protein sequences we noticed great sequence diversities among the nine in the transcriptome. I-antigen proteins in *C*. *irritans* are surface membrane proteins without known functions, and have been used as antigens to immunize fish against *C*. *irritans* infections [20, 21, 23]. To help elucidate the phylogenetic relationship among I-antigen sequences, we searched through the NCBI protein database using BLAST and identified 18 more *C*. *irritans* I-antigen sequences (S2 Table). Together with the nine I-antigen sequences published by Lokanathan et al. [24], 27 I-antigen sequences were subjected to phylogenetic analysis using a maximum-likelihood method [38]. We found that these 27 sequences were separated into at least three major groups, judging by their genetic distances (Fig 6). The two sequences with the longest genetic distance, GEEV01000054.1 and GEEV01000039.1, shared only 49.4% in sequence identity and 62.7% in similarity. Despite great sequence diversities, there were conserved motifs that could be detected in 24 full-length I-antigen sequences (the other three were incomplete or truncated). There were also 11 conserved cysteine sites which may be of future interest to study the function of I-antigen proteins (S3 Fig).

## Discussion

How a parasite finds and successfully infects its hosts presents a unique challenge to the parasite itself. Without the nutrient-providing host, a parasite would have limited energy supplies before finding hosts and establishing new infections. In its theront form, despite showing an oral apparatus [41–43], it is generally assumed that *C*. *irritans* either does not feed or undergoes limited feeding from the aquatic environment and therefore cannot meet its energy demands. The limited-energy-supplies assumption is supported by observations made by Yoshinaga and Dickerson [29], who showed that 81% of theronts lost mobility as early as 8.5 hrs post-excystment. A recent study that quantitatively described theront movements also showed a much narrower range of movements in theronts collected 12 hrs post excystment [44]. Our RNA-seq results seem to corroborate with these physiological observations: many genes involved in ATP-producing processes are significantly downregulated in aging theronts (Fig 5 and Table 3).

On the other hand, the loss of food sources and/or energy supplies may not be the sole factor contributing to the loss of infectivity or the death of *C*. *irritans* theronts. Chu et al. showed that when providing homogenized fish tissues to theront cells, their averaged survival period was significantly shortened compared to the control group kept in the sterile sea water. More interestingly, theronts stayed active longer in the Medium Essential Medium Eagle (MEME) medium than in sterile sea water, but died very quickly when provided with non-essential amino acids in the MEME medium [28]. Clearly, theronts respond to various environmental cues differently and further research is necessary to help discover what triggers corresponding responses in theront cells.

Our results suggest that aging theronts seem to translate more new proteins as genes involved in transcription/translation processes are upregulated 10 hrs post excystment (Fig 5A and S8 Table). However, we did not find any transcription factor genes that were significantly upregulated or downregulated. This may be due to a low number of transcription factor genes present in the moderate size of transcriptome. In addition, the upregulation/downregulation of regulatory genes might have occurred between our two sampling time points and we simply missed it.

Expression of the anti-stress gene, Bax1 inhibitor, was also elevated at 10hrs. With a limited transcriptome we were unable to further identify what clusters of genes aging theronts might need to express and ascertain the implications. It is possible that *C*. *irritans* theronts are programmed to start expressing genes needed for the trophont transformation at a late time point. An equally plausible explanation is that aging theronts may be activating salvaging and/or anti-stress pathways to stay alive until a host is found. Further analyses are needed when a well-annotated genome or a clean, more comprehensive *C*. *irritans* transcriptome becomes available.

One other consistent pattern that we observed was the downregulation of the 9 I-antigen genes (8 of them were DEGs) in the *C*. *irritans* transcriptome we used. I-antigens are outer membrane surface GPI-linked proteins with unknown functions capable of inducing immune responses in host fish [20–23]. Several attempts have been made to immunize fish with either recombinant I-antigen proteins [20, 23] or in the form of DNA vaccine [21], with different degrees of immune protection. Using BLAST we identified 9 I-antigen transcripts in the transcriptome published by Lokanathan and colleagues [24]. This number is consistent with the number of I-antigen transcripts reported in another *C*. *irritans* transcriptome study [26], suggesting that *C*. *irritans* might indeed have 9 active I-antigen genes. These I-antigen sequences are, however, quite diverse: the most diverged pair of sequences share only 49.4% in sequence identity and 62.7% similarity. Results derived from our phylogenetic analyses on 27 published I-antigen sequences reveal that these I-antigens can be separated into at least three distinct subgroups (Fig 6). Interestingly, only sequences in one of the three groups have been tested for vaccination (sequences with asterisks in Fig 6) [20, 21, 23]. Given that I-antigen genes in another group (GEEV01000094, 95, and 96) were also highly expressed (Table 3 and S8 Table) in young theronts it may be worth testing whether the use of this group of sequences, and/or in combination with other sequences, could help achieve better vaccination results.

The synchronous downregulation of the expression of all 9 I-antigen genes indicates that these genes may be under the control of similar promoters and/or transcription factors. Most I-antigen transcripts were detected in the tomont stage [26], although I-antigen proteins were found on the surface of theronts [23] and antibodies produced by fish against I-antigens conferred immunological protection [20–23, 45]. Taken together, most I-antigen genes in *C*. *irritans* might first be expressed in the tomont stage at a high level for tomont maturation and that expression gradually fade away in theront and trophont stages. Interestingly, in the distantly related parasitic ciliate *Ichthyophthirius multifiliis*, which causes the white-spot-disease in freshwater fish and homoplastically exhibits three similar morphostages: trophonts, tomonts, and theronts, I-antigen transcripts were abundant in theronts and trophonts [46, 47]. It is likely that I-antigens in *C*. *irritans and I*. *multifiliis* play different roles and are under different developmental controls, despite the fact that I-antigens in both species are GPI-linked surface proteins and are strong immunogen sources [48–51].

Finally, the downregulation of four V-ATPase genes is of particular interest. V-ATPase genes are highly conserved across eukaryotes and V-ATPase is responsible for acidifying membrane-bound organelles, such as the lysosome and vacuole, by a proton pumping-rotary mechanism [52]. The complete protein complex consists of two domains: the water soluble ATPase domain (V_1_) and the membrane integrated proton pump domain (V_O_). V_1_ is composed of subunits A-H while V_O_ is composed of subunits a, c, d, and e. Our results show that the expression of subunits A, C, E, and d were significantly downregulated in 10 hrs theronts (Table 3). Furthermore, the A (alpha) subunit of V-ATPase was detected in the theront, but not in trophont or tomont stages in a recent proteomic study [25]. Taking these observations together the expression of V-ATPase might indeed be stage-specific. However, in a presumably non-phagocytic stage what are the target vacuoles for the V-ATPase, and how this protein complex affects the infectivity of *C*. *irritans* remain to be elucidated.

## Supporting information

S1 Fig Plot of *C*. *irritans* gene expression values from 1 hr and 10hrs theront cells. Log FPKM values, derived from RNA-seq data, of genes that were expressed either or both in 1hr and 10 hrs theront cells are plotted against each other (R^2^ = 0.844).
(PDF)

S2 Fig Level 6 GO term distributions among significantly (a) upregulated and (b) downregulated genes (PPDE> = 0.95).
(PDF)

S3 Fig T-coffee alignment of 24 complete I-antigen sequences. Despite their high diversities, there are conserved sequences denoted with * (fully conserved),: (highly conserved), and. (semi conserved) in the cons row. Colors reflect quality of the alignment from high (pink) to low (blue).
(PDF)

S1 Table Primer information. (DOCX)

S2 Table GenBank accession numbers of *C*. *irritans* I-antigen sequences. (DOCX)

S3 Table BLAST results from unique transcripts with higher than 42% G/C. (XLSX)

S4 Table List of top 100 highly expressed genes. (PDF)

S5 Table Information of significantly regulated genes (PPDE > = 0.95). (XLSX)

S6 Table BLAST results from differentially expressed genes. (XLSX)

S7 Table List of cellular modification proteins. (XLSX)

S8 Table Information of differentially expressed genes. (XLSX)

S9 Table GO term breakdowns of proteins with ATP-binding domain. (XLSX)

S10 Table Genes related to regulation of gene expression. (XLSX)
