# Supplementary figures and images for: Infectivity and genes differentially expressed between young and aging theront cells of the marine fish parasite Cryptocaryon irritans
Source: PLoS One. 2020 Aug 28;15(8):e0238167. doi: 10.1371/journal.pone.0238167 (PMC7454944; doi:10.1371/journal.pone.0238167)

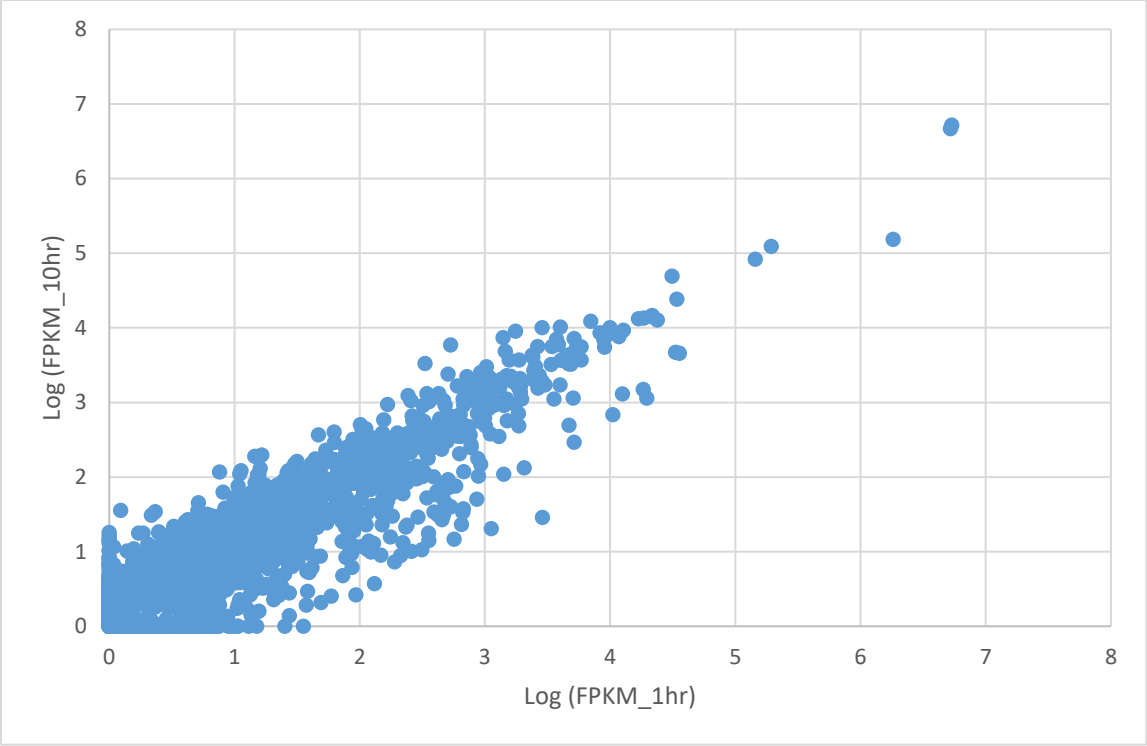

Supplement: S1 Fig — Log FPKM values, derived from RNA-seq data, of genes that were expressed either or both in 1hr and 10 hrs theront cells are plotted against each other (R2 = 0.844). (PDF) [file pone.0238167.s001.pdf]

(a)

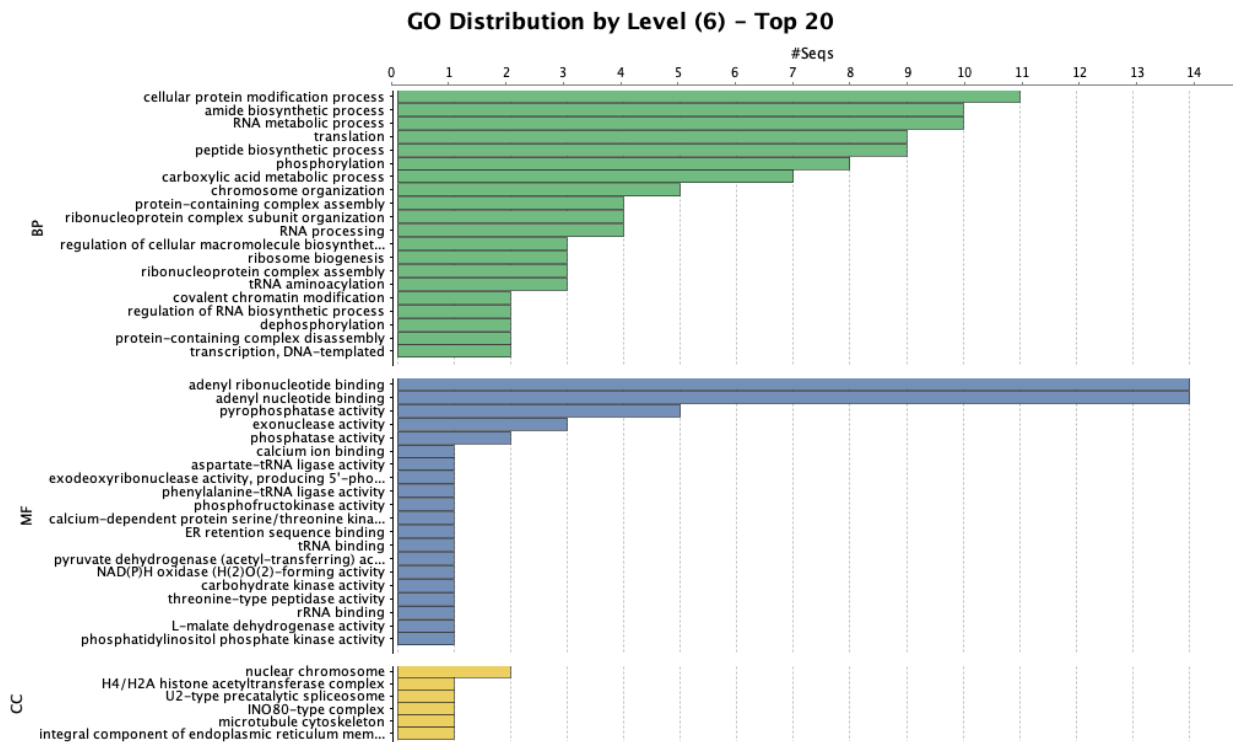

(b)

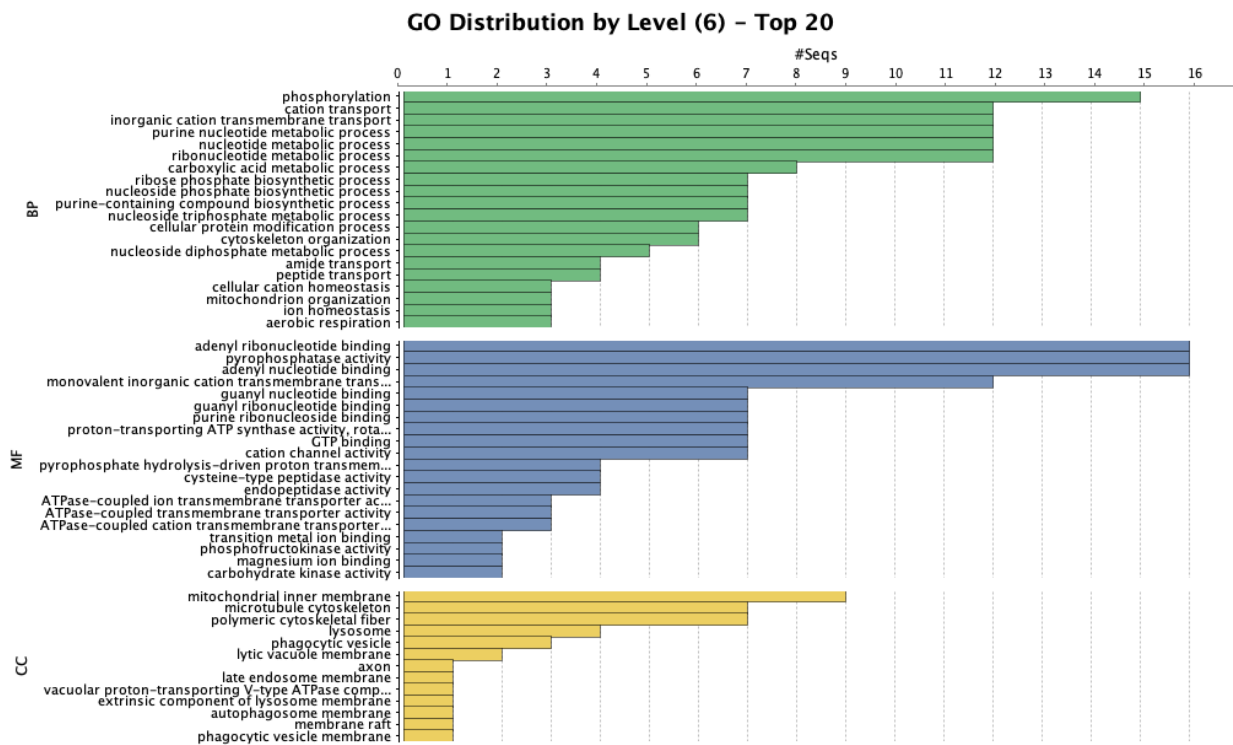

Supplement: S2 Fig — Level 6 GO term distributions among significantly (a) upregulated and (b) downregulated genes (PPDE> = 0.95). (PDF) [file pone.0238167.s002.pdf]
