# Supplementary material for: Infectivity and genes differentially expressed between young and aging theront cells of the marine fish parasite Cryptocaryon irritans
Source: PLoS One. 2020 Aug 28;15(8):e0238167. doi: 10.1371/journal.pone.0238167 (PMC7454944; doi:10.1371/journal.pone.0238167)
Supplement: S3 Fig — Despite their high diversities, there are conserved sequences denoted with * (fully conserved),: (highly conserved), and. (semi conserved) in the cons row. Colors reflect quality of the alignment from high (pink) to low (blue). (PDF) [file pone.0238167.s003.pdf]

```
T-COFFEE, Version_11.00.8cbe486 (2014-08-
12 22:05:29 - Revision 8cbe486 - Build 477)
Cedric Notredame
CPU TIME:0 sec.
SCORE=966
*
 BAD AVG GOOD
*
AB262047        :   96
AB381932        :   95
ACN89782        :   96
ACN89783        :   96
ADZ38984        :   96
ADZ38985        :   96
AEE39297        :   94
AEH21941        :   96
AGA16539        :   96
AGK30041        :   96
AWQ64538        :   96
BAF37969        :   96
BAF37970        :   96
BAF37971        :   96
BAF37972        :   96
BAF37973        :   96
BAG16623        :   95
GEEV01000037    :   96
GEEV01000038    :   96
GEEV01000039    :   96
GEEV01000053    :   94
GEEV01000054    :   94
GEEV01000095    :   95
GEEV01000096    :   95
cons            :   96

AB262047        MQKIISILLISSLAVMTSAAWAPKTAAADWKGTFVVTKSSCLATCGWK
AB381932        MSKFIIALLIASFAILNQCAWVEKTAVADWTGTFIVVKASCSASCSYK
ACN89782        MQKILAILLISSLAVITSAAFVKKTAAADWKGTFVVTKSSCLATCGWK
ACN89783        MQKILAILLISSLAVISSAAWAEKTAVADWKGTFVVTKSSCLATCGWK
ADZ38984        -------------------------AVADWTGTFIVVKATCSASCSYK
ADZ38985        -------------------------AVADWTGTFIVVKASCADSCSYK
AEE39297        -------------------------AVADWTGTFVVTKSSCNQYCGWK
AEH21941        MQKILAILLISSLAVISSAAWAEKTAVADWKGTFVVTKSSCLATCGWK
AGA16539        MQKILAILLISSLAVISSAAWAEKTAIADWKGTFVVTKSSCLTTCGWK
AGK30041        MQKIIAILLISSLAVISSAAWAEKTAIADWKGTFVVTKSSCLATCGWK
AWQ64538        MQKILAILLISSLAVISSAAWAEKTAVADWKGTFVVTKSSCLATCGWK
BAF37969        MQKIISILLISSLAVMTSAAWAPKTAAADWKGTFVVTKSSCLATCGWK
BAF37970        ------------------AAWAPKTAAADWKGTFVVTKSSCLATCGWK
BAF37971        ---------ISSLAVMTSAAWAPKTAAADWKGTFVVTKSSCLATCGWK
```

```
BAF37972       ---------ISSLAVMTSAAWAPKTAAADWKGTFVVTKSSCLATCGWK
BAF37973       ---------ISSLAVMTSAAWAPKTAAADWKGTFVVTKSSCLATRGWK
BAG16623       MSKFIIALLIASFAILNQCAWVEKTAVADWTGTFIVVKASCSASCSYK
GEEV01000037   ----LAILLISSLAVFASANWVEKTAAADWKGTFVVTSSSCLASCGWK
GEEV01000038   ----VAILLISSFVAISSAAWVAKTTAADWKGTFVVTSSSCLATCGWK
GEEV01000039   ----LAILLISSLAVISSAAWAEKTAVADWKGTFVVTKSSCLATCGWK
GEEV01000053   -----IALLVASLTISTQAAFTAVTAMADWKGSFVVTKSTCNQYCGWK
GEEV01000054   --------------------------MADWKGTFVVTKSSCNQYCGWK
GEEV01000095   -----IALLIASFALLNQCAWVEKTTVADWTGTFIVVKASCSASCSYK
GEEV01000096   ---------IASFALLNHCAFVEKTTVADWTGTFIVVKASCADSCSYK

cons                                      ***.*:*:*..::*    .:*


AB262047       IGTTIVIADKTGD-SSKVTWMGTVHTTDSTNVDV-VSGSCKYVSVVAT
AB381932       LGSEIKITPTTGATTTKTTWAGTAHSTDSTDVTV-ASGTCKYVTSVAS
ACN89782       LGSTVVIADKTGV-NTKVTWVGTTHTTDTTNVDV-AAGSCKYISAVTT
ACN89783       IGSTIVIAEKTGD-NTKVTWVGTVHTTDATNVDV-ASGSCKYVSVVAA
ADZ38984       LGSEIKITATTGATTTKTTWAGTAHTTDSTNVDV-ASGSCKYVSAVTN
ADZ38985       LGTEVKITETAGSTSTKTTWAGTAHTTDSTNVDV-ASGSCKYVSAVAN
AEE39297       VGTQVVITEKT---ATVATWTGTTFTSDTTNAEITAAGVCQYAKEKTA
AEH21941       IGSTIVIAEKTGD-NTKVTWVGTVHTTDATNVDV-ASGSCKYVSVVAA
AGA16539       IGSTIVIAEKSGD-NTKATWVGTVHTTDATNVDV-ASGSCKYVCVVAT
AGK30041       IGTTIVIAEKSGD-NTKATWVGSVHTTDATNVDV-ASGSCKYVSVVAT
AWQ64538       IGSTIVIAEKTGD-NTKATWVGTVHTTDATNVDV-ASGSCKYVSVVAA
BAF37969       IGTTIVIADKTGD-SSKVTWMGTVHTTDSTNVDV-VSGSCKYVSVVAT
BAF37970       IGTTIVIADKTGD-SSKVTWMGTVHTTDSTNVDV-VRGSCKYVSVVAT
BAF37971       IGTTIVIADKSGD-NTKVTWMGTVHTTDSTNVDV-TSGSCKYVSVVAT
BAF37972       IGTTIVIADKSGD-NTKVTWMGTVHTTDSTNVDV-TSGSCKYVSVVAT
BAF37973       IGTTIVIADKSGD-NTKVTWVGTVHTTDSTNVDV-TSGSCKYVSVVAT
BAG16623       LGSEIKITPTTGATTTKTTWAGTAHSTDSTDVTV-ASGTCKYVTSVAS
GEEV01000037   IGTTVVIADKASD-ATKVTWVGTAHTTDSTNVDV-ASGSCKYVSAVAN
GEEV01000038   IGTTVVIADKTGD-ATKVTWQGTVHTTDTTNVDV-ATGSCKYVSIVAT
GEEV01000039   IGSTIVIAEKTGD-NTKATWVGTVHTTDATNVDV-ATGSCKYVSVVAA
GEEV01000053   LGSQVDITEKS---TTVATWTGTTFTSDTTNAEVTTAGACQYAKEKGS
GEEV01000054   VGTQVVITEKT---ATVATWTGTTFTSDTTNAEITAAGVCQYAKEKTA
GEEV01000095   LGSEIKITATTATPTTKTTWAGTVISSDTTDVTV-ASGVCSYVTSVAG
GEEV01000096   LGTEVKITETAGSTSTKTTWAGTASSTDSTDVTV-ASGKCSYVTSVTS

cons           :*: : *: .:    : .** *:. ::*:*:. : . * *.*


AB262047       AGTAGTPAEVLKNSDECAFATGTCGIMGRKQSTPGTVTFNRDTTLDTK
AB381932       -GTPAAAADILKDADECVVATGICTTQGQKQTTAGKINFKRDMDMATK
ACN89782       AGQAGTPAEVANNNDECEFASGTCTVMGRKQTTPGTVVFNRDMDLDTK
ACN89783       AGTPGTAAEVLKNDDECVFATGMCTIMGQKQTKPAKITFNRDTTLDTK
ADZ38984       AGTAGTAAEVLNSNDECVFATGMCTIMGQKQKSPAKVTFNRDTTLDTK
```

```
ADZ38985        AGTPGTAAEVLNNNDECAFASGVCTVMGQKQKTPAKVTFKRDTTLDTK
AEE39297        --AAIAKVDILSKTDDCTIATGACVTMGQKAATTANTEFKRDTTVATK
AEH21941        AGTPGTAAEVLKNDDECVFATGMCTIMGQKQTKPAKITFNRDTTLDTK
AGA16539        AGTAGTPAEVLKNDDECTVASGICTIMGQKQTTPKKVTFNRDTALDTK
AGK30041        AGTAGTAAEVLKNDDECIVGTGICTIMGQKQTTPKKVTFNRDTALDTK
AWQ64538        AGTPGTAAEVLKNDDECVVGTGMCTIMGQKQTKPAKITFNRDTALDTK
BAF37969        AGTAGTPAEVLKNSDECAFATGTCGIMGRKQSTPGTVTFNRDTTLDTK
BAF37970        AGTAGTPAEVLKNSDECAFATGTCGIMGRKQSTPGTVTFNRDTTLDTK
BAF37971        AGTAGTPAEVLKNSDECAFATGTCSIMGRKKATPGTVTFNRDMTLDTK
BAF37972        AGTAGTPAEVLKNSDECAFATGTCSIMGRKKATPGTVTFNRDMTLDTK
BAF37973        AGTAGTPAEVLKNSDECAFATGTCSIMGRKKATPGTVTFNRDMTLDTK
BAG16623        -GTPAAAADILKDADECVVATGICTTQGQKQTTAGKINFKRDMDMATK
GEEV01000037    AGTPGTAAEVLNNNDECAFASGVCTVMGQKQKTPAKVTFKRDTTLDTK
GEEV01000038    EGQVGTATEVLNNDDTCEFGNGACTVMGKKQKTPGVVAFKRDMDLDTK
GEEV01000039    AGTPGTAAEVLKNDDECVVGTGMCTIMGQKQTKPAKITFNRDTALDTK
GEEV01000053    --TAIAKVDILTKTDDCTISSGACTVMGQKAVTAANTEFKRDKALATK
GEEV01000054    --AAIAKVDILSKTDDCTIATGACVTMGQKAATTANTEFKRDTTVATK
GEEV01000095    -GTPAAAAEVLKDADECTVATGICTVQGQKQTSAGKINFKRDMDLATK
GEEV01000096    -GTADAAVDILNDADECTVATGVCTNQGQKQTTKGKINFKRDMDLATK

cons                 : .:: .. * * ...* *   *:*  .     *:**  : **


AB262047        PLQILYKQFAMVQKTSTTQKATATDQAANCDTQASFVDTTTDAKAIVG
AB381932        PFQTTYKQLKVIKPKTSTTNAAAADQTADCVTEADMVDTALDAKDIVG
ACN89782        PLQILYKQFEMIAKSSTSVKA-AADQGADCDTQASLVDITTDAKPIVG
ACN89783        PLQVLYKQFEMVQKSSTTQKAAATDQAADCDTQASMVDTTTDAKAIVG
ADZ38984        PFQILYKQLAMVPKAQTTVQA-AADQATDCDTKASLVDTTTDAKAIVG
ADZ38985        PFQILYKQLAMVPKAQTTVQA-AADQAADCDTQASLVDTTTDAKAIVG
AEE39297        PLQITYPQWTLLPLT-TSTATAASDIAAKCITEADMVDTTVDVKGLSG
AEH21941        PLQVLYKQFEMVQKSSTTQKAAATDQAADCDTQASMVDTTTDAKAIVG
AGA16539        PLQILYKQFDMVAKTSTTQKAAATDQATDCDTQASMVDTTTDAKAIVG
AGK30041        PLQILYKQFEMVAKTSTAQKTSATDQAADCDTQASMVDTTTDAKAIVG
AWQ64538        PLQVLYKQFEMVQKSSTQQKATATDQAADCDTQASMVDTTTDAKAIVG
BAF37969        PLQILYKQFAMVQKTSTTQKATATDQAANCDTQASFVDTTTDAKAIVG
BAF37970        PLQILYKQFAMVQKTSTTQKATATDQAANCDTQASFVDTTTDAKAIVG
BAF37971        PFQILYKQFEMVQKTSTTQKATATDQAADCDTQASFVDITTDAKAIVG
BAF37972        PFQILYKQFEMVQKTSTTQKATATDQAADCDTQASFVDITTDAKAIVG
BAF37973        PFQILYKQFEMVQKTSTTQKATATDQAADCDTQASFVDITTDAKAIVG
BAG16623        PFQTTYKQLKVIKPKTSTTNAAAADQTADCVTEADMVDTALDAKDIVG
GEEV01000037    PFQILYKQLAMVPKAQTTVQA-AADQAADCDTQASLVDTTTDAKSIVG
GEEV01000038    PFQILYKQVEMVKKTSTTQKAAATDQANDCDTQASLVDTTTDAKAIVG
GEEV01000039    PLQVLYKQFEMVPKSSTQQKATATDQAADCDTQASMVDTTTDAKAIVG
GEEV01000053    PLQITYPQWQLVALS-TTATTNAADIVAKCITEADMIDTTVDVKDLAG
GEEV01000054    PLQITYPQWTLLPLT-TSTATAASDIAAKCITEADMVDTTVDVKGLSG
GEEV01000095    PFQTTYKQWVVIKPKTSTVNTAASDQGTECVTEADMIDTALDAKDIVG
GEEV01000096    PFQTTYKQWKVIKPKTSTTTAAAADQTADCVTEAEMIDTTLDAKDIVG
```

```
cons            *:*  * *  ::    :   : *:*   .* *:*.::* : *.* : *


AB262047        SLKLSKASCDKCSWDTTKDLKITQ-DASKKYMVTLAGTIK-ETATGDC
AB381932        TLKLTEAACGSCSWDTSKELKISQHDSSKKYMVTLAGTIKGKSAVGDC
ACN89782        TLKLSKATCDKCSWDITKDLKITQ-DATNKYMVTLAGTIK-ETATGDC
ACN89783        TLKLSKAVCNKCSWDTTKDLKITQ-DATNKYMVTLAGTLK-ETATGDC
ADZ38984        TLKLSKAVCNKCSWDTTKDLKITQ-DATNKYMVTLAGTLK-ETATGDC
ADZ38985        TLKLSKATCDKCSWDTTKDLKITQ-DATNKYMVTLAGTIK-ETNTGDC
AEE39297        TVTLTSASCGTCTWDSTKTLKITQ-DSTKKYMVKLEGTIK-ENPAGSC
AEH21941        TLKLSKAVCNKCSWDTTKDLKITQ-DATNKYMVTLAGTLK-ETATGDC
AGA16539        TLKLSKAVCNKCSWDTTKDLKITQ-DATNKYMVTLAGTLK-ETATGDC
AGK30041        TLKLSKAACNKCSWDTTKDLKITQ-DATNKYMVTLAGTLK-ETATGDC
AWQ64538        TLKLSKAVCNKCSWDTTKDLKITQ-DATNKYMVTLAGTLK-ETATGDC
BAF37969        SLKLSKASCDKCSWDTTKDLKITQ-DASKKYMVTLAGTIK-ETATGDC
BAF37970        SLKLSKASCDKCSWDTTKDLKITQ-DASKKYMVTLAGTIK-ETATGDC
BAF37971        TLKLSKASCDNCSWDTTKDLKITQ-NASKKYMVTLAGTIK-ETATGDC
BAF37972        TLKLSKASCDNCSWDTTKDLKITQ-NASKKYMVTLAGTIK-ETATGDC
BAF37973        TLKLSKASCDKCSWDTTKDLKITQ-NASKKYMVTLAGTIK-ETATGDC
BAG16623        TLKLTEAACGSCSWDTSKELKISQHDSSKKYMVTLAGTIKGKSAVGDC
GEEV01000037    TLKLSKATCDKCSWDTTKDLKITQ-DATKKYMVTLAGTIK-ETTSGDC
GEEV01000038    TLKLSKATCDKCSWDTSKDLKITQ-DATKKYMVTLAGTIK-ETTAGDC
GEEV01000039    TLKLSKAVCNKCSWDTTKDLKITQ-DATNKYMVTLAGTLK-ETATGDC
GEEV01000053    TVTLTSATCGTCTWDSTKTLKITQ-DSTKKYMVKLEGTLK-ESTTGSC
GEEV01000054    TVTLTSASCGTCTWDSTKTLKITQ-DSTKKYMVKLEGTIK-ENPAGSC
GEEV01000095    TLKLSEAACGSCSWDITKELKISQHDSAKKYMVTLAGTIKGKTGASDC
GEEV01000096    TLKLSEAACGTCSWDTSKELKISQDDSTKKYMVTLAGTIKGKSAASDC

cons            ::.*:.* *..*:** :* ***:* ::::****.* **:* :.  ..*


AB262047        NNKLTASEACYVTKKD-DKTYVLVSCATLDTTSKGIPIAITTANSKTT
AB381932        TNKLTATEKCYAAKKD-ANNWAVFGCTTLQSPVGGIPIVKATASGKTT
ACN89782        KNKLTASETCYVTKKD-DKTFILVSCTTLDTTGSGIPIVISTVNSKTT
ACN89783        KDKLTASEKCYVTKKD-DKTYILVSCTTLDTTNSGIPIVIATVSSKTT
ADZ38984        KDKLTASEKCYVTKKD-DKTYILVSCTTLDTTNSGIPIVIATVSSKTT
ADZ38985        KNKLTASETCYVTKKD-DKTFILVSCTTLDTTGSGIPIVISTVNSKTT
AEE39297        TGKLTNAENCHAIKDSTAEKYYLYGCTTLWTPTGGIPITLTTASGKTT
AEH21941        KDKLTASEKCYVTKKD-DKTYILVSCTTLDTTNSGIPIVIATVSSKTT
AGA16539        KDKLTASEKCYVTKKD-DKTYILVSCTTLDTTNSGIPIVISTVSSKTT
AGK30041        KDKLTASEKCYVTKKD-DKTYILVSCTTLDTTNSGIPIVITTVSSKTT
AWQ64538        KDKLTASEKCYVTKKD-DKTYILVSCTTLDTTNSGIPIVIATVSSKTT
BAF37969        NNKLTASEACYVTKKD-DKTYVLVSCATLDTTSKGIPIAITTANSKTT
BAF37970        NNKLTASEACYVTKKD-DKTYVLVSCATLDTTSKGIPTAITTANSKTT
BAF37971        NNKLTASEACYVTKKD-DKTYVLVSCATLDTTSKGIPIAITTANSKTT
BAF37972        NNKLTASEACYVTKKD-DKTYVLVSCATLDTTSKGIPIAIATANSKTT
```

```
BAF37973         NNKLTASEACYVTKKD-DKTYVLVSCATLDTTSKGIPIAITTANSKTT
BAG16623         TNKLTATEKCYAAKKD-ANNWAVFGCTTLQSPVGGIPIVKATASGKTT
GEEV01000037     KNKLTASEACYVTKKD-DKTFILVSCTTLDTTNKGIPIAITTVNSKTT
GEEV01000038     ANKLTASETCYVTKKD-DKTYILVSCTTLDTTNKGIPIAITTVSSKTT
GEEV01000039     KDKLTASEKCYVTKKD-DKTYILVSCTTLDTTNSGIPIVIATVSSKTT
GEEV01000053     TGKLTTAENCHAIKDSTANKYYLYGCTTLWTPTGGIPATLATASGKTT
GEEV01000054     TGKLTNAENCHAIKDSTAEKYYLYGCTTLWTPTGGIPITLTTASGKTT
GEEV01000095     TNKLTDTEKCYAAKKD-ANNWAVFGCTTLQSPTGGIPIVKATVSSKTT
GEEV01000096     TNKLTNTEKCYAAKKD-ANNWAVFGCTTLQSPLGGIPIVKATVSSKTT

cons              .*** :* *:. *..  :.: : .*:** :.  *** . :*...***


AB262047         LTMTWTDSASKACSVVGEVTSS--SNSVKLISGFSAMLILSLALLFK
AB381932         LTMAWQ-SGSDNCKVVGEVTSTSGSNALRYISNISMMLIIALALLFK
ACN89782         LTLTWKDATAQACNVVGEVSSTSGANSLKLFTGLSVMLILTFALLFK
ACN89783         LTMTWTDSGSNACSVVGEVTSTSGSISLKMFTGFSAMLILAFALLFK
ADZ38984         LTMTWTDSGSNACNVVGEVTSSSGSISLKMFTGFSAMLILALALLFK
ADZ38985         LTLTWKDATAQACNVVGEVSSTSGANSLKLFTGLSVMLILTLALLFK
AEE39297         LSMSWSDSASTACKVEGSFAAGTTTNAIKIFSGLSMMLLLALALLFK
AEH21941         LTMTWTDSGSNACSVVGEVTSTSGSISLKMFTGFSAMLILAFALLFK
AGA16539         LTMAWTDSTSNACSVVGEVTSSSGSVSLKMFTGFSAMLILAFALLFK
AGK30041         LTMTWTDSASNACSVVGEVTSTSGSISLKMFTGLSAMLILALALLFK
AWQ64538         LTMTWTDSGSNACNVVGEVTSSSGSISLKMFTGFSAMLILAFALLFK
BAF37969         LTMTWTDSASKACSVVGEVTSS--SNSVKLISGFSAMLILSLALLFK
BAF37970         LTMTWTDSASKACSVVGEVTSS--SNSVKLISGFSAMLILSLALLFK
BAF37971         LTMTWTDSASKACSVVGEVSSS--SNSVKLISGFSAMLILSFALLFK
BAF37972         LTMTWTDSASKACSVVGEVSSS--SNSVKLISGFSAMLILSFALLFK
BAF37973         LTMTWTDSASKACSVVGEVSSS--SNSVKLISGFSAMLILSFALLFK
BAG16623         LTMAWQ-SGSDNCKVVGEVTSTSGSNALRYISNISMMLIIALALLFK
GEEV01000037     LTLTWTDSASQACNVVGEITSTSGSNSLKMFSGLSAMIIIAFAILFK
GEEV01000038     LTLTWTDSAAKACNVVGEVTSTSGSNSLKILSGFSAMLILAFALLYK
GEEV01000039     LTMTWTDSGSNACNVVGEVTSSSGSISLKMFTGFSAMLILAFALLFK
GEEV01000053     LTLEWSDSASAACKVEGSFAAGGTTNALKMISGLSMMLLLALSLLLK
GEEV01000054     LSMSWSDSASTACKVEGSFAAGTTTNAIKIFSGLSMMLLLALGLLFK
GEEV01000095     YTLTWT-SGSDNCKVVGEFTSGSGSNAFKLFSGLSMMLVIALGLLFK
GEEV01000096     YTLAWT-SGSDNCKVVGEFTSGS-SSALKIFSGFSMMLVIALSILFR

cons              :: *  : :  *.* *..::   : :.: ::.:* *:::::.:* :
```
