# Supplementary material for: Infectivity and genes differentially expressed between young and aging theront cells of the marine fish parasite Cryptocaryon irritans
Source: PLoS One. 2020 Aug 28;15(8):e0238167. doi: 10.1371/journal.pone.0238167 (PMC7454944; doi:10.1371/journal.pone.0238167)
Supplement: S1 Table — (DOCX) [file pone.0238167.s004.docx]

| Name | Sequence | Strand | Position | Tm °C | Purification method | Modification |
| --- | --- | --- | --- | --- | --- | --- |
| 18S ribosomal RNA gene | | | | | | |
| 18S-F1 | GCGCGCTATACTGACACATT | forward | 1413 | 55.94 | PAGE |  |
| 18S-R1 | AAAGGGCAGGGACGTAATCA | reverse | 1563 | 56.01 | PAGE |  |
| 18S-P1 | ACCCGAAACCCTTCCGGGCCA | reverse | 1448 | 65.30 | HPLC | 5'Fam - 3'Tamra |
| gb_GEEV01000050.1_guanine nucleotide-binding subunit beta | | | | | | |
| GN-F1 | TTCGCTAGTTGTGGATGGGA | forward | 554 | 55.75 | PAGE |  |
| GN-R1 | AGCTGGAGCTCAAATCGGTA | reverse | 721 | 55.83 | PAGE |  |
| GN-P1 | ACTTTGTCTTTGCCTCCAGTGGCAAG | reverse | 677 | 62.11 | HPLC | 5'Fam - 3'Tamra |
| gb_GEEV01002455.1_kinase domain | | | | | | |
| KD-F1 | CCAGAATATCTGGCTCCCGAA | forward | 559 | 56.17 | PAGE |  |
| KD-R1 | TGGAGGTAACCCAGTACACA | reverse | 647 | 54.58 | PAGE |  |
| KD-P1 | TCCACCAGTCTACAGCCTTGCCA | reverse | 600 | 62.15 | HPLC | 5'Fam - 3'Tamra |
| gb_GEEV01001290.1_eukaryotic initiation factor 4A isoform X1 | | | | | | |
| 1290-F1 | GACCCTGCTTCAAGTCACAC | forward | 226 | 55.87 | PAGE |  |
| 1290-R1 | GGTTCCTCCTGTGCAAGAAT | reverse | 338 | 54.83 | PAGE |  |
| 1290-P1 | CTCGCTCCAACTAGAGAGCTGGCT | forward | 259 | 62.26 | HPLC | 5'Fam - 3'Tamra |
| gb_GEEV01000496.1_inhibitor of apoptosis-promoting Bax1 | | | | | | |
| BI1-F1 | CAATTCAGCAGCTTCGGCTT | forward | 99 | 56.57 | PAGE |  |
| BI1-R1 | AAGGCGAGACCACTTGAACA | reverse | 214 | 56.57 | PAGE |  |
| BI1-P1 | TGCACCTGCAACAGAGGCACC | reverse | 172 | 62.62 | HPLC | 5'Fam - 3'Tamra |
| gb_GEEV01000568.1_probable gtpase rab7 | | | | | | |
| GR-F1 | CCACTGTTGAGCTTTCGCTT | forward | 268 | 56.16 | PAGE |  |
| GR-R1 | GAGGAGCTGATTGCTGTGTG | reverse | 442 | 56.02 | PAGE |  |
| GR-P1 | CGGGATCCTTTGGAGCACCCTGCA | forward | 351 | 65.26 | HPLC | 5'Fam - 3'Tamra |
| gb_GEEV01000318.1_eukaryotic initiation factor 4A isoform X1 | | | | | | |
| 318-F1 | TCTTGCACAGGAGGAACAAA | forward | 379 | 54.30 | PAGE |  |
| 318-R1 | ACCAAGCATTTCATCTGCTTCA | reverse | 525 | 55.56 | PAGE |  |
| 318-P1 | CAGCGACTCTCCCTGGAGTTCCA | reverse | 447 | 62.24 | HPLC | 5'Fam - 3'Tamra |
| gb_GEEV01000060.1_enolase | | | | | | |
| Enolase-F1 | TCGTCTTGCCTGTTCCTTCA | forward | 430 | 56.28 | PAGE |  |
| Enolase-R1 | TGGTAAACCTCAGCACCGAT | reverse | 552 | 56.03 | PAGE |  |
| Enolase-P1 | TGTTACCGGCATGCTTTCCTCCATTG | reverse | 461 | 62.05 | HPLC | 5'Fam - 3'Tamra |
| gb_GEEV01002316.1_solute carrier family 12 member 4 isoform X4 | | | | | | |
| 2316-F1 | AGATCTTTAGGGCCCGCTTT | forward | 303 | 56.07 | PAGE |  |
| 2316-R1 | TCCTGTCCATGTTAATCCAGT | reverse | 414 | 53.17 | PAGE |  |
| 2316-P1 | ACATAGCAGCTCCACAAGCATTAGCCA | reverse | 352 | 62.69 | HPLC | 5'Fam - 3'Tamra |
| gb_GEEV01000479.1_carbohydrate binding partial | | | | | | |
| CB-F1 | TGCAATAGAGGTGCAGCAAA | forward | 271 | 55.14 | PAGE |  |
| CB-R1 | TCATGCCAAATGTGCTATTATCT | reverse | 428 | 52.96 | PAGE |  |
| CB-P1 | ACCTCCCATTCCATGCTCTGATTCCAG | reverse | 332 | 62.29 | HPLC | 5'Fam - 3'Tamra |
| gb_GEEV01000654.1_autophagy 3 | | | | | | |
| A3-F1 | TACGCAGTCTTGTCCAACTT | forward | 147 | 54.16 | PAGE |  |
| A3-R1 | TTCGGGAGGAAATGCAGGAT | reverse | 203 | 56.07 | PAGE |  |
| A3-P1 | AGCTGCCGGCTTCCACTGCC | reverse | 167 | 64.97 | HPLC | 5'Fam - 3'Tamra |
| gb_GEEV01001025.1_6-phosphofructokinase | | | | | | |
| PFK1-F1 | GGGCTGATACTGCAATCAGAG | forward | 256 | 55.43 | PAGE |  |
| PFK1-R1 | TGTGTCATAGGCATTAGCTGGA | reverse | 434 | 55.92 | PAGE |  |
| PFK1-P1 | CCCTGAGGCATCTGTTCCACCTTGCA | reverse | 289 | 64.83 | HPLC | 5'Fam - 3'Tamra |
| gb_GEEV01002446.1_ATP synthase beta subunit | | | | | | |
| 2446-F1 | TGGTTTGGTTAGAGAATAGCCAGT | forward | 307 | 56.12 | PAGE |  |
| 2446-R1 | AGGAGCGTCCCTATGAATGG | reverse | 450 | 55.97 | PAGE |  |
| 2446-P1 | ACGGGAGGCCCTATCATGGTTCC | forward | 338 | 62.40 | HPLC | 5'Fam - 3'Tamra |
| gb_GEEV01000007.1_vacuolar ATP synthase subunit A | | | | | | |
| 07-F1 | GGCTGCTAGAGAAGCCTCAA | forward | 944 | 56.50 | PAGE |  |
| 07-R1 | CTGCATCAGCTGGCATTTCA | reverse | 1082 | 56.26 | PAGE |  |
| 07-P1 | TGCCCATCGTGATGTAGAATCTGCCA | reverse | 1024 | 62.34 | HPLC | 5'Fam - 3'Tamra |
| gb_GEEV01000842.1_transcription factor | | | | | | |
| TF-F1 | ATTCGGAGTTCAGCCTTTGC | forward | 198 | 55.91 | PAGE |  |
| TF-R1 | CAGGCAGCAACTCCTTGAAA | reverse | 342 | 55.74 | PAGE |  |
| TF-P1 | TGGTGTCGGCATGACCCATCACT | reverse | 315 | 62.76 | HPLC | 5'Fam - 3'Tamra |
| gb_GEEV01000449.1_citrate synthase | | | | | | |
| CS-F1 | TGTTGCCATAGATTTAAGAAGAAGGT | forward | 85 | 55.04 | PAGE |  |
| CS-R1 | AGCCTCTGTATGTTAATCCAAGT | reverse | 230 | 53.86 | PAGE |  |
| CS-P1 | AGCAATGGAGCTCCTCCCATTCCA | reverse | 176 | 62.21 | HPLC | 5'Fam - 3'Tamra |
| gb_GEEV01000053.1_agglutination immobilization antigen | | | | | | |
| iAg-F1 | TGGACTCCAACTGGAGGTAT | forward | 771 | 54.32 | PAGE |  |
| iAg-R1 | ACTTTGCAAGCAGCACTAGC | reverse | 852 | 56.51 | PAGE |  |
| iAg-P1 | TCCAGCTACCCTTGCCACAGCA | forward | 791 | 62.82 | HPLC | 5'Fam - 3'Tamra |
