# Supplementary material for: Infectivity and genes differentially expressed between young and aging theront cells of the marine fish parasite Cryptocaryon irritans
Source: PLoS One. 2020 Aug 28;15(8):e0238167. doi: 10.1371/journal.pone.0238167 (PMC7454944; doi:10.1371/journal.pone.0238167)
Supplement: S2 Table — (DOCX) [file pone.0238167.s005.docx]

**S3 Table.** GenBank Accession Numbers of *C. irritans* I-Antigen Sequences

GEEV01000054.1, GEEV01000053.1, GEEV01000036.1, GEEV01000037.1, GEEV01000038.1, GEEV01000039.1, GEEV01000094.1, GEEV01000095.1, GEEV01000096.1, AGA16539, AEE39297, AB381932, BAG16623, ADZ38985, ADZ38984, AEH21941, ACN89783, AWQ64538, AGK30041, AEC12209, ACN89782, BAF37973, BAF37972, BAF37971, BAF37970, BAF37969, AB262047.
