# Supplementary material for: Infectivity and genes differentially expressed between young and aging theront cells of the marine fish parasite Cryptocaryon irritans
Source: PLoS One. 2020 Aug 28;15(8):e0238167. doi: 10.1371/journal.pone.0238167 (PMC7454944; doi:10.1371/journal.pone.0238167)
Supplement: S4 Table — (PDF) [file pone.0238167.s007.pdf]

S2 Table. List of Top 100 Expressed Genes

1hr	10hr
gb|GEEV01000049.1|	gb|GEEV01000049.1|
gb|GEEV01000189.1|	gb|GEEV01000189.1|
gb|GEEV01000137.1|	gb|GEEV01000137.1|
gb|GEEV01000838.1|	gb|GEEV01000838.1|
gb|GEEV01000435.1|	gb|GEEV01000435.1|
gb|GEEV01000095.1|	gb|GEEV01000100.1|
gb|GEEV01000542.1|	gb|GEEV01000542.1|
gb|GEEV01000094.1|	gb|GEEV01000392.1|
gb|GEEV01000100.1|	gb|GEEV01000279.1|
gb|GEEV01000231.1|	gb|GEEV01000148.1|
gb|GEEV01000392.1|	gb|GEEV01001835.1|
gb|GEEV01000279.1|	gb|GEEV01000231.1|
gb|GEEV01000502.1|	gb|GEEV01001253.1|
gb|GEEV01000148.1|	gb|GEEV01000050.1|
gb|GEEV01000074.1|	gb|GEEV01000406.1|
gb|GEEV01001835.1|	gb|GEEV01001074.1|
gb|GEEV01001419.1|	gb|GEEV01001419.1|
gb|GEEV01000096.1|	gb|GEEV01001492.1|
gb|GEEV01000327.1|	gb|GEEV01000041.1|
gb|GEEV01000039.1|	gb|GEEV01001776.1|
gb|GEEV01001074.1|	gb|GEEV01000327.1|
gb|GEEV01001776.1|	gb|GEEV01000077.1|
gb|GEEV01000551.1|	gb|GEEV01000020.1|
gb|GEEV01000611.1|	gb|GEEV01001084.1|
gb|GEEV01000041.1|	gb|GEEV01000611.1|
gb|GEEV01001253.1|	gb|GEEV01000293.1|
gb|GEEV01000330.1|	gb|GEEV01000248.1|
gb|GEEV01000495.1|	gb|GEEV01001560.1|
gb|GEEV01000450.1|	gb|GEEV01000970.1|
gb|GEEV01000038.1|	gb|GEEV01000495.1|
gb|GEEV01000020.1|	gb|GEEV01000551.1|
gb|GEEV01001488.1|	gb|GEEV01000450.1|
gb|GEEV01000609.1|	gb|GEEV01000429.1|
gb|GEEV01001255.1|	gb|GEEV01000094.1|
gb|GEEV01000377.1|	gb|GEEV01000095.1|
gb|GEEV01000037.1|	gb|GEEV01000377.1|
gb|GEEV01000472.1|	gb|GEEV01002027.1|
gb|GEEV01002189.1|	gb|GEEV01000947.1|
gb|GEEV01000947.1|	gb|GEEV01001255.1|
gb|GEEV01000274.1|	gb|GEEV01001126.1|
gb|GEEV01002540.1|	gb|GEEV01002189.1|
gb|GEEV01000050.1|	gb|GEEV01000274.1|
gb|GEEV01002153.1|	gb|GEEV01000330.1|
gb|GEEV01000293.1|	gb|GEEV01000415.1|
gb|GEEV01001084.1|	gb|GEEV01001723.1|

gb|GEEV01000899.1|	gb|GEEV01002540.1|
gb|GEEV01001560.1|	gb|GEEV01000702.1|
gb|GEEV01000055.1|	gb|GEEV01000472.1|
gb|GEEV01001722.1|	gb|GEEV01000609.1|
gb|GEEV01000364.1|	gb|GEEV01000055.1|
gb|GEEV01000406.1|	gb|GEEV01000352.1|
gb|GEEV01000230.1|	gb|GEEV01001618.1|
gb|GEEV01000515.1|	gb|GEEV01000353.1|
gb|GEEV01000266.1|	gb|GEEV01002590.1|
gb|GEEV01001941.1|	gb|GEEV01000833.1|
gb|GEEV01000970.1|	gb|GEEV01000515.1|
gb|GEEV01000352.1|	gb|GEEV01000516.1|
gb|GEEV01000353.1|	gb|GEEV01000828.1|
gb|GEEV01000251.1|	gb|GEEV01002554.1|
gb|GEEV01001126.1|	gb|GEEV01001865.1|
gb|GEEV01002027.1|	gb|GEEV01001472.1|
gb|GEEV01000054.1|	gb|GEEV01002559.1|
gb|GEEV01001428.1|	gb|GEEV01001299.1|
gb|GEEV01002610.1|	gb|GEEV01001751.1|
gb|GEEV01001674.1|	gb|GEEV01001641.1|
gb|GEEV01001299.1|	gb|GEEV01001510.1|
gb|GEEV01000415.1|	gb|GEEV01000251.1|
gb|GEEV01000141.1|	gb|GEEV01001962.1|
gb|GEEV01000493.1|	gb|GEEV01000230.1|
gb|GEEV01000134.1|	gb|GEEV01000211.1|
gb|GEEV01002108.1|	gb|GEEV01000907.1|
gb|GEEV01001492.1|	gb|GEEV01000863.1|
gb|GEEV01001514.1|	gb|GEEV01001939.1|
gb|GEEV01001962.1|	gb|GEEV01002271.1|
gb|GEEV01000863.1|	gb|GEEV01000426.1|
gb|GEEV01001641.1|	gb|GEEV01002108.1|
gb|GEEV01000828.1|	gb|GEEV01001077.1|
gb|GEEV01001723.1|	gb|GEEV01001722.1|
gb|GEEV01000318.1|	gb|GEEV01002153.1|
gb|GEEV01002507.1|	gb|GEEV01000140.1|
gb|GEEV01000516.1|	gb|GEEV01000266.1|
gb|GEEV01000429.1|	gb|GEEV01000233.1|
gb|GEEV01002344.1|	gb|GEEV01001674.1|
gb|GEEV01000667.1|	gb|GEEV01000695.1|
gb|GEEV01000077.1|	gb|GEEV01000141.1|
gb|GEEV01002114.1|	gb|GEEV01001941.1|
gb|GEEV01001510.1|	gb|GEEV01001596.1|
gb|GEEV01001077.1|	gb|GEEV01000074.1|
gb|GEEV01000695.1|	gb|GEEV01000944.1|
gb|GEEV01000143.1|	gb|GEEV01002437.1|
gb|GEEV01000270.1|	gb|GEEV01002610.1|
gb|GEEV01002271.1|	gb|GEEV01001316.1|

gb|GEEV01000211.1|	gb|GEEV01001610.1|
gb|GEEV01002437.1|	gb|GEEV01002423.1|
gb|GEEV01000053.1|	gb|GEEV01001018.1|
gb|GEEV01002559.1|	gb|GEEV01000096.1|
gb|GEEV01001721.1|	gb|GEEV01001757.1|
gb|GEEV01001610.1|	gb|GEEV01000443.1|
gb|GEEV01001043.1|	gb|GEEV01002604.1|
gb|GEEV01001399.1|	gb|GEEV01000822.1|
